# Comprehensive Analysis and Functional Studies of WRKY Transcription Factors in *Nelumbo nucifera*

**DOI:** 10.3390/ijms20205006

**Published:** 2019-10-10

**Authors:** Jing Li, Yacen Xiong, Yi Li, Shiqi Ye, Qi Yin, Siqi Gao, Dong Yang, Mei Yang, E. Tapio Palva, Xianbao Deng

**Affiliations:** 1 School of Chemistry, Chemical Engineering and Life Sciences, Wuhan University of Technology, Wuhan 430070, China; jinli@whut.edu.cn (J.L.); xiongyacen@whut.edu.cn (Y.X.); ly247295@whut.edu.cn (Y.L.); yeshiqi@whut.edu.cn (S.Y.); yyqq@whut.edu.cn (Q.Y.);; 2 Key Laboratory of Plant Germplasm Enhancement and Specialty Agriculture, Wuhan Botanical Garden, Chinese Academy of Sciences, Wuhan 430074, China; yangdong@wbgcas.cn (D.Y.); yangmei815815@wbgcas.cn (M.Y.); 3 Center of Economic Botany, Core Botanical Gardens, Chinese Academy of Sciences, Wuhan 430074, China; 4 Viikki Biocenter, Department of Biosciences, Division of Genetics, University of Helsinki, 00100 Helsinki, Finland; tapio.palva@helsinki.fi

**Keywords:** WRKY, Lotus, transcription factor, evolution, benzylisoquinoline alkaloids

## Abstract

The WRKY family is one of the largest transcription factor (TF) families in plants and plays central roles in modulating plant stress responses and developmental processes, as well as secondary metabolic regulations. Lotus (*Nelumbo nucifera*) is an aquatic crop that has significant food, ornamental and pharmacological values. Here, we performed an overview analysis of WRKY TF family members in lotus, and studied their functions in environmental adaptation and regulation of lotus benzylisoquinoline alkaloid (BIA) biosynthesis. A total of 65 *WRKY* genes were identified in the lotus genome and they were well clustered in a similar pattern with their *Arabidopsis* homologs in seven groups (designated I, IIa-IIe, and III), although no lotus WRKY was clustered in the group IIIa. Most lotus WRKYs were functionally paired, which was attributed to the recently occurred whole genome duplication in lotus. In addition, lotus *WRKYs* were regulated dramatically by salicilic acid (SA), jasmonic acid (JA), and submergence treatments, and two lotus WRKYs, NnWRKY40a and NnWRKY40b, were significantly induced by JA and promoted lotus BIA biosynthesis through activating BIA biosynthetic genes. The investigation of WRKY TFs for this basal eudicot reveals new insights into the evolution of the *WRKY* family, and provides fundamental information for their functional studies and lotus breeding.

## 1. Introduction

The WRKY family is among the 10 largest transcription factor (TF) families in higher plants and is found throughout the green lineage [1]. The WRKY TFs derive their name from their characteristic WRKY DBD, which comprises approximately 60 amino acids and a highly conserved sequence of WRKYGQK in the N-terminal together with a novel C2H2 or C2HC zinc finger motif in the C-terminal. Besides the core sequence of WRKYGQK, there are also several other heptapeptide variants, such as WRKYGKK, WKKYGQK, WRKYGRK, WRKYGEK and WSKYEQK [2,3]. Both the WRKY and the Zinc finger motif are believed to be crucial for proper DNA binding [4]. Based on the number of WRKY domains and the features of the zinc finger motifs, WRKY TFs are classified into three distinct groups. Those harboring two WRKY domains and a C2H2-type zinc finger motif belong to WRKY group I, while those harboring one WRKY domain but with different types of zinc finger motif fall into WRKY groups II and III. Group II is further divided into five subgroups designated IIa, IIb, IIc, IId and IIe based on the amino acid motifs outside the core WRKY domain and their phylogenetic relationship [5,6,7,8]. The conservation of the *WRKY* gene family was also shown by its high binding affinity to W box (C/TTGACT/C) [5,9], although altered binding preference to WK box (TTTTCCAC) and WT box (GGACTTTC) have been reported [10,11,12].

WRKY TFs were originally thought to be plant-specific [5], but were later also found in other organisms, such as diplomonads, amoebae, fungi, incertae sedis, and amoebozoa [6,13]. Those non-plant WRKY TFs, however, do not belong to any of the three WRKY groups found in flowering plants, and are assumed to have originated from a number of ancient gene transfer events [13]. It is proposed that *WRKY* gene family in early eukaryotes may have been derived from a single copy, and expansion of the gene family has resulted mainly from tandem and/or segmental gene duplications, and gain or loss of domains [1,6,13]. However, these evolution models of the *WRKY* gene family are expected to vary as far as more organisms are considered [8,14].

Since the first report of a WRKY protein in sweet potato [15], significant advances have been made to elucidate their regulatory functions. WRKY TFs play significant roles in regulating plant responses to various biotic and abiotic stresses [16,17,18,19,20]. During these responsive processes, phytohormones, such as salicylic acid (SA), jasmonic acid (JA) and abscisic acid (ABA) are important signaling molecules associated with the WRKY-mediated resistance [3,21]. In addition, WRKY TFs also control various physiological and developmental processes, including embryogenesis, seed germination, trichome development, flowering modulation, fruit ripening, and leaf senescence, as well as secondary metabolic regulation [22,23,24,25,26,27]. 

Increased availability of plant genome sequences and the implementation of relevant bioinformatics tools have enabled the genome-wide identification of *WRKY* genes in many plant species, including at least 79 crop species, as well as many important plant models [14]. The genome of *Amborella trichopoda*, the most recent common ancestor of all flowering plants, encodes 32 *WRKY* genes [28], which is significantly fewer than that in the plant model *Arabidopsis*, which encodes 72–74 *WRKY* genes [5,29]. In contrast, most crop species contain more *WRKY* genes due to their large and complex genomes. For example, the soybean (*Glycine max*) genome encodes as many as 182 *WRKY* genes, while rice (*Oryza sativa*) and cotton (*Gossypium hirsutum*) genomes encode 98 (or 102) and 102 WRKY members, respectively [30,31,32].

Lotus (*Nelumbo nucifera*) is a perennial aquatic plant that has significant ornamental and food values. It is also a traditional herb both in China and India. All parts of the lotus plant have been used for treating various diseases [33]. Modern pharmacological studies have confirmed its activities against cancer, diabetes, obesity, hypertension, and HIV, and found that alkaloids were the most relevant bioactive component in lotus [34]. To date, 46 alkaloid compounds have been isolated from various lotus organs, 45 of which belong to the class of benzylisoquinoline alkaloid (BIA) [34]. Our previous studies have shown that lotus BIAs accumulate primarily in lotus leaf and embryo [35], and the biosynthesis of lotus BIA was transcriptionally regulated [36]. It has been reported that the overexpression of *WRKYs* could activate a set of BIA biosynthetic genes and significantly enhance the BIA production in *Coptis japonica* and *California poppy* [25,26]. Consistently, six lotus WRKYs also appeared in the list of candidate regulators of lotus BIA biosynthesis [36]. Here we report a comprehensive analysis of WRKY transcription factors in lotus and studies of their functions in response to various stresses and in lotus BIA biosynthesis.

## 2. Results

### 2.1. Identification of WRKY Family Members

To identify WRKY encoding genes in lotus genome, coding DNA sequences (CDS) of all *Arabidopsis WRKY* genes (*AtWRKY*s) were used as queries to search lotus *WRKY* homologues (*NnWRKYs*) in the HMMER web server (http://hmmer.janelia.org). After removing overlaps and those without WRKY domains, a total of 65 *NnWRKY* genes were identified from the lotus genome of ‘China Antique’ (ASM303368v1) [37]. These *NnWRKY* genes are unevenly distributed in the eight lotus chromosomes, with 16 genes on chromosome 1 (Chr 1), and 11, 11, 8, 8, 5, 5 genes on chromosomes 2, 3, 4, 5, 6, 8, respectively, while only a single gene was identified on Chr 7 (Table S1). The open reading frame of the *NnWRKY* genes varied from 312 to 2694 bp, and their deduced protein length ranged from 104 to 898 amino acids (Table S1).

The 65 NnWRKY proteins were named by their predicted functions after two-round inspections (Table S1). First, all NnWRKY protein sequences were used as queries to search against the lotus genome database using the phmmer program of the HMMER web server. Most NnWRKYs retrieved their functional names with an E value of approximately 1 × e-100. Subsequently, all WRKYs, individually, were manually blasted against both the *Arabidopsis* and the lotus genome databases. In case two or three NnWRKYs share high sequence similarity, and were annotated to the same functional name, lower case letters a, b, or c were added at the end of their functional names to distinguish them. Of the genes identified, 56 were found to be functionally in pairs, three formed a triad (NnWRKY40a, NnWRKY40b, and NnWRKY40c), while the remaining six were in singletons. High similarity in both nucleic and amino acid sequences was observed between paired WRKY homologues in lotus (Table 1). Lotus gene *NNU_24697* is a paralog of the singleton *NnWRKY9*, and show highly similarity also with *AtWRKY9* (Figure S1). However, it does not harbor a WRKY domain, thus was not included in the NnWRKY list. Two NnWRKYs, NNU_04803 and NNU_23618, were un-named due to their unknown predicted functions, and without both lotus and *Arabidopsis* homologues.

Of the 65 NnWRKY proteins, 59 contain the conserved WRKYGQK heptapeptide, two (NnWRKY51a and NnWRKY51b) contain the WRKYGKK domain, and two (NnWRKY50a and NnWRKY50b) contain the WKKYGKK domain. Interestingly, the two NnWRKY proteins NNU_04803 and NNU_23618 with unknown functions contain novel WRKY domains, as WRKYSEK and WKKYGQK respectively (Table S1). In addition, all identified NnWRKYs contained the conserved zinc finger domain (C2H2 or C2HC) except for NnWRKY17b, which had a missing C-terminal zinc finger domain.

### 2.2. Phylogenetic Analysis of NnWRKY Family Genes

**Figure 1:**
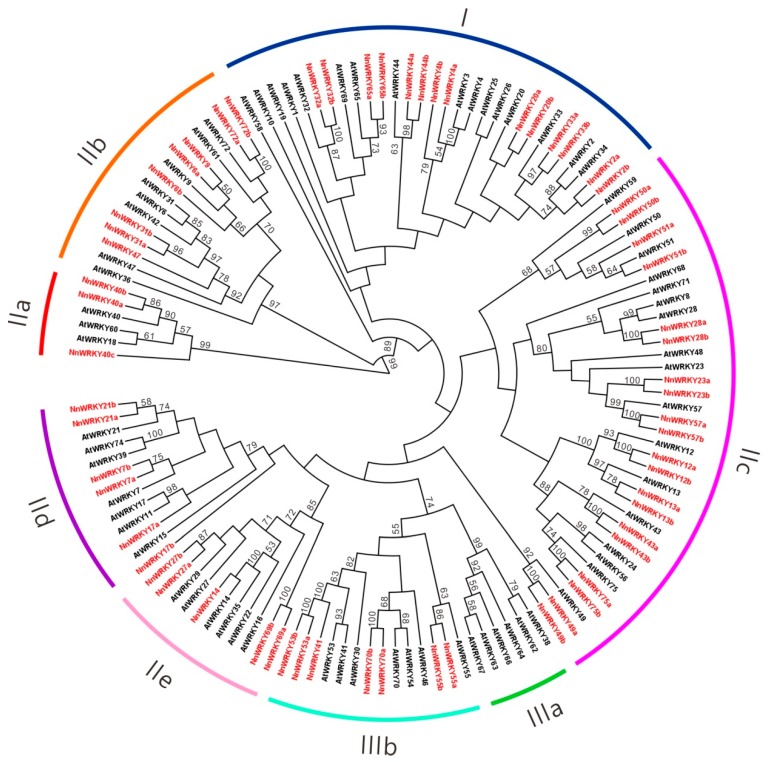
Phylogenetic tree of lotus (*Nelumbo nucifera*) and *Arabidopsis thaliana* WRKY proteins. The tree is based on the full-length protein sequences of 63 lotus and 70 *Arabidopsis* WRKYs shown in red and black respectively. Amino acid sequences were aligned with MUSCLE, and the phylogenetic tree was constructed using the neighbor-joining method in MEGA7 with 1000 bootstrap replications. Arcs with different colors represent different WRKY clusters according to *Arabidopsis* classifications.

In order to verify the functions of NnWRKYs, a neighbor-joining phylogenetic tree was constructed based on the full-length protein sequences of 63 NnWRKYs and 70 AtWRKYs. Together with *Arabidopsis* homologues, NnWRKYs were well clustered into seven groups, in accordance with the previous report by Eulgem et al. [5]. Group I contains 14 NnWRKYs clustered in seven pairs, while group III contains seven NnWRKYs. Subgroups IIa, IIb, IIc, IId and IIe each contain 3, 8, 20, 6, and 5 NnWRKYs, respectively (Figure 1). Consistent with their predicted functions, NnWRKYs were grouped together with their *Arabidopsis* homologues, and nearly all NnWRKY pairs (or triads) were grouped together with significantly high branch values. Most NnWRKYs clustered in group I (11 of 14) contain two WRKY domains, while the NnWRKY65a and WRKY65b only contain one WRKY domain, similar to their *Arabidopsis* homologue AtWRKY65, and NnWRKY33a has obviously lost its C-terminal WRKY domain in comparison to NnWRKY33b (Figure S2). NnWRKYs in group II and III only contain one WRKY domain, but harbor a different type of zinc finger motifs. NnWRKYs in subgroup II contain the C2H2 type zinc finger motif, while NnWRKYs in group III are of the C2HC type. It is worth noting that there was no lotus WRKY assigned to the phylogenetic subgroup IIIa (Figure 1).

### 2.3. NnWRKY Gene Structures and Conserved Motifs

**Figure 2:**
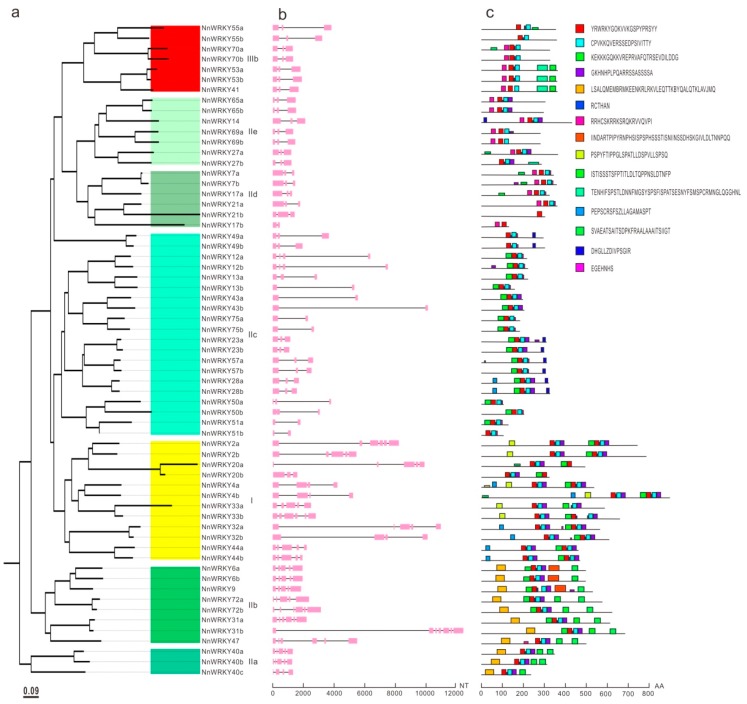
Gene structures and conserved motifs of *NnWRKY* genes. (**a**) The neighbor-joining tree is based on the full-length protein sequences of 63 lotus WRKYs, and was constructed in MEGA7 with 1000 bootstrap replications. Different phylogenetic clusters are marked with different colors. (**b**) The exon–intron structures of *NnWRKY* genes were analyzed using the online Gene Structure Display Server (GSDS, http://gsds.cbi.pku.edu.cn), and the exons and introns are marked with pink blocks and black lines, respectively. (**c**) Conserved motifs were predicted using the MEME online program (http://meme-suite.org/tools/meme), and are marked with colored boxes.

For a better understanding of their potential functions, *NnWRKY* gene structures and their conserved protein motifs were analyzed. The number of exons and introns in the *NnWRKYs* varies from 2 to 7, with 31 (47.7%) *NnWRKY*s containing 3 exons, 12 (18.5%) *NnWRKY*s containing 5 exons, and with 8, 6, 5, 1 *NnWRKY*s containing 2, 4, 6, and 7 exons respectively (Figure 2a,b). Paired *NnWRKY*s tend to have rather similar coding sequence lengths and equal number of exons. *NnWRKY*s in group IId, IIe and IIIb, for example, all contain 3 exons, except for the *NnWRKY17b*, which contains only 2 exons due to the loss of its C-terminal zinc finger motif. Similarly, *NnWRKY*s in group IIc contain 2–3 exons, whereas *NnWRKY*s in group I, IIa, and IIb tend to contain 4 to 7 exons.

Similar to the patterns observed in *NnWRKY* gene structures, NnWRKYs in the same phylogenetic group tend to contain similar conserved motifs. Many paired NnWRKYs share the same conserved motifs. For example, paired NnWRKY53a and NnWRKY53b, NnWRKY65a and NnWRKY65b, NnWRKY49a and NnWRKY49b, NnWRKY13a and NnWRKY13b, NnWRKY43a and NnWRKY43b, and NnWRKY75a and NnWRKY75b all share very similar conserved motifs. A total of 15 conserved motifs were discovered by the MEME program. Of these, the four motifs around the WRKY and the zinc finger domains were the mostly conserved (Figure 2c).

### 2.4. Synteny Analysis of NnWRKY Genes

**Figure 3:**
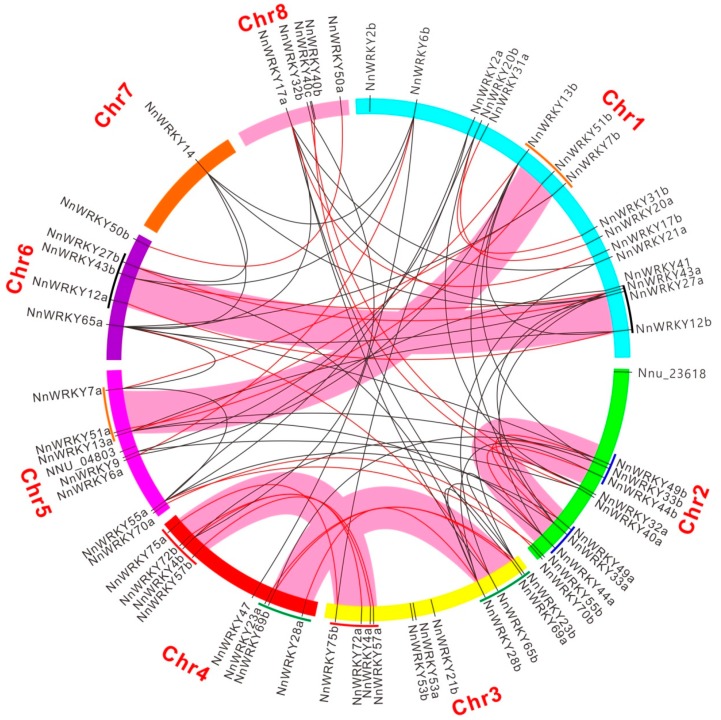
Syntenic relationship of *NnWRKY* genes. All 65 genes are marked according to their localization on lotus chromosomes. Functionally paired syntenic genes are connected with red lines, and others are connected with black lines. Chromosome areas, in which more than two lotus *WRKY* genes showed collinearity in the same order, are connected with thick pink lines.

To understand the evolutionary divergence of *NnWRKYs*, we performed an intragenomic synteny analysis among *NnWRKYs*. Each *NnWRKY* gene was individually used as a query to search for *NnWRKY* syntelogs based on the SynFind program in the CoGe platform [38]. As a result, intensive synteny among *NnWRKYs* was observed (Figure 3). Query genes from one phylogenetic group mostly targeted *WRKY* syntelogs of the same group (Figure S3). A group IIc member *NnWRKY23b*, for example, found a total of five syntenic *NnWRKY* genes, four of which were located in the same group. Intergroup syntenies between IId and IIe, I and IIc, IIa and IIb were relatively strong, suggesting a more recent divergence of the members between those groups. In contrast, syntenies between members of groups IIIb and IIa, and IIIb and IIb were hardly detected, indicating a much earlier divergence between the *WRKY* genes in those groups.

Of the 28 *NnWRKYs* pairs (Table 1), 25 paired *NnWRKY*s were found to be syntenic to each other (Figure 3). This indicated that these duplicated *NnWRKY* pairs might be derived from whole genome duplication (WGD) events or other chromosome segmental duplications, which further confirmed their paralogy. Two gene pairs, *NnWRKY53a* and *NnWRKY53b*, and *NnWRKY40b* and *NnWRKY40c*, were tandemly arrayed duplicates on Chr 3 and 8 respectively. *NnWRKY40b* and *NnWRKY40c* are also syntenic with their *NnWRKY40a* homologue. However, two gene pairs, *NnWRKY2a* and *NnWRKY2b*, and *NnWRKY21a* and *NnWRKY21b*, were not syntenic to each other. These two pairs of *NnWRKYs* may be derived by small scale gene translocation events. Among the six singleton *NnWRKYs*, four (*NnWRKY9*, *NnWRKY14*, *NnWRKY41*, and *NnWRKY47*) targeted one to four syntelogs, while the remaining two (*NNU_04803* and *NNU_23618*) with unknown functions found no syntelog. 

**Figure 4:**
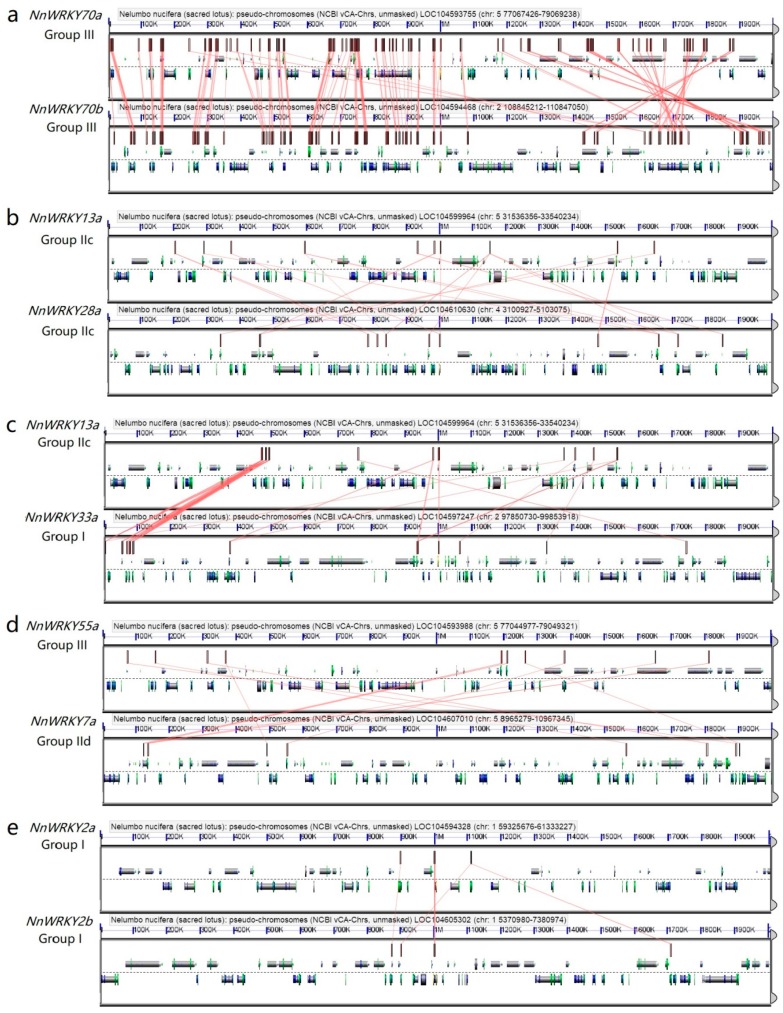
Microsynteny analysis for representative lotus *NnWRKY* genes. (**a**) functionally paired genes with syntenic relationship; (**b**) syntenic genes within group IIc; (**c**) syntenic genes from group IIc and group I, respectively; (**d**) syntenic genes from group III and group IId, respectively; and (**e**), functionally paired genes with no syntenic relationship.

We also conducted microsynteny analysis within a 2-Mbp region for selected syntenic and non-syntenic *NnWRKYs*. As a result, strong collinearity was observed between functionally paired *NnWRKY70a* and *NnWRKY70b*, with 137 paralogue gene pairs found in the compared region (Figure 4a, connected with red lines; the magnified version of Figure 4 is shown in Figure S4), which is in accordance with above synteny analysis. Weak chromosomal collinearity was observed among un-paired syntenic genes within the same phylogenetic group, such as *NnWRKY13a* and *NnWRKY28a* (Figure 4b), and between phylogenetic groups, such as *NnWRKY13a* and *NnWRKY33a* (Figure 4c), and *NnWRKY55a* and *NnWRKY7a* (Figure 4d). By contrast, functionally paired but non-syntenic *NnWRKYs*, for example, *NnWRKY2a* and *NnWRKY2b*, showed the least collinearity, with only four paralogue gene pairs found in the analyzed chromosome area (Figure 4e). Intriguingly, our syntenic analysis among *NnWRKYs* also revealed five possible big chromosomal duplication events (shown in pink thick lines, Figure 3). That is, big chromosomal regions harboring several *NnWRKYs* were duplicated at a time and still remain conserved in their current location.

### 2.5. Spatial Expression Patterns of NnWRKYs

**Figure 5:**
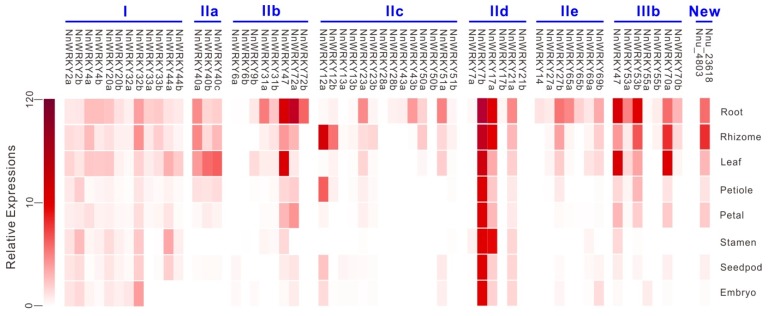
Spatial expression patterns of *NnWRKY* genes. Samples were taken from lotus root, rhizome, leaf, petal, stamen, seedpod, and embryo, respectively, and relative expressions of lotus *WRKY* genes were evaluated with real-time PCR. The color represents average expression of three biological replicates for each sample. Lotus *WRKY* genes from the same phylogenetic group are grouped together.

To explore possible physiological functions of *NnWRKY*s, we performed real-time PCR, and checked their spatial expression patterns in various lotus organs including root, rhizome, leaf, petiole, petal, stamen, seedpod and embryo. Most *NnWRKY*s, except for *NNU_04803*, *NnWRKY21b*, and *NnWRKY28a*, were obviously expressed in at least one of the eight tested organs (Figure 5). In addition, *NnWRKY*s seemed to be more preferentially expressed in root, rhizome and leaf. Despite showing diverse expression patterns, similar profiles were observed for *NnWRKY* genes from the same phylogenetic group. For example, group IIa members were mainly expressed in root, rhizome, leaf and petiole, but were nearly undetectable in other organs. Each of the three gene pairs in group IId had only one gene expressed in all eight organs, whereas the other member was almost un-detectable (Figure 5). Of the 65 tested *NnWRKY* genes, *NnWRKY7b* showed relatively high expression in eight different organs, and the highest expression in the root tissue, suggesting its fundamental role in plant growth and development.

### 2.6. Responses of NnWRKYs to SA, JA, and Submergence Treatments

**Figure 6:**
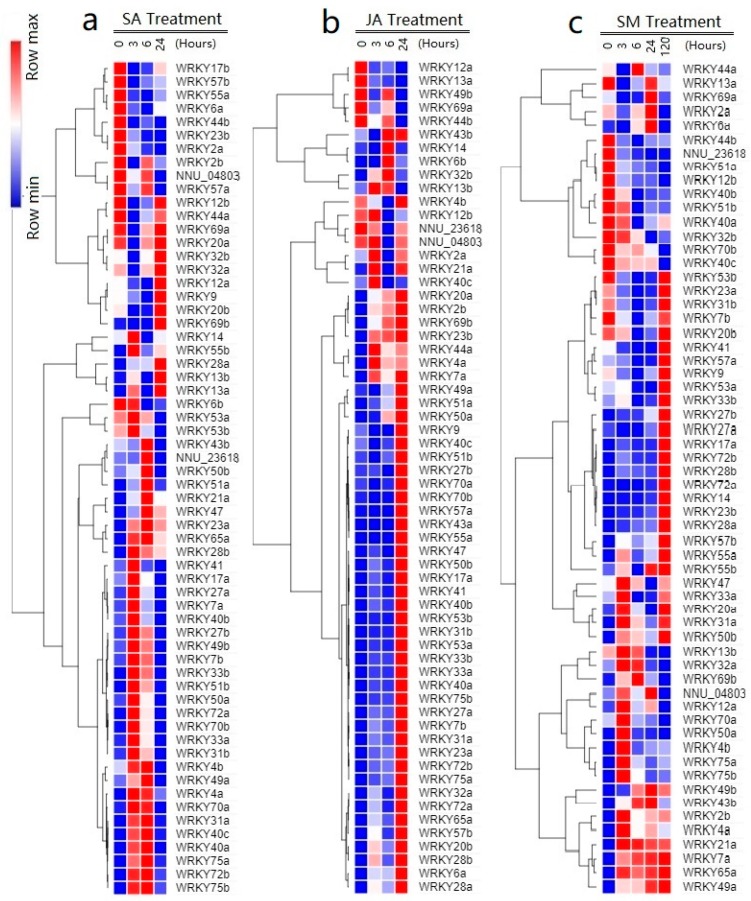
Expression profiles of lotus *WRKY* genes under the SA (**a**), JA (**b**) and submergence (**c**) treatment. The heatmap was generated with the online program, Morpheus (https://software.broadinstitute.org/morpheus/), and the digital expression data from RNA sequencing assay were gene-wise normalized and hierarchical clustered with average linkage in rows.

To investigate potential roles of NnWRKYs in mediating plant defense to various stresses, responses of *NnWRKYs* to hormone treatments and environmental stimuli were examined. *NnWRKY* genes responded rapidly after SA treatment (Figure 6a). A total of 25 and 6 *NnWRKYs* were significantly up- and down-regulated respectively at 3 h post treatment (hpt), indicating their association with the SA-mediated signaling pathway. These up-regulated *NnWRKYs*, however, tend to revert back to their initial expression levels at 24 hpt (Figure 6a). Three of the six initially down-regulated genes also returned to their original expression levels at 24 hpt, whereas the remaining three remained at lower expression levels. Interestingly, seven *NnWRKYs* showed late response to SA treatment, with gene expression significantly up-regulated at 6 hpt or 24 hpt. In comparison to SA treatment, responses of *NnWRKYs* to JA treatment were more consistent. A set of 24 *NnWRKYs* were significantly up-regulated at 3 hpt, all of which were maintained at high expression levels until 24 hpt (Figure 6b). In addition, 10 *NnWRKYs* were significantly up-regulated late at 24 hpt, while three *NnWRKYs* were down-regulated after JA treatment.

A more complex response of *NnWRKYs* to submergence treatment was observed (Figure 6c). A total of 23 *NnWRKYs* were significantly up-regulated at 3 hpt, 16 of which remained at high expression levels until 120 hpt. Nine *NnWRKYs* were significantly down-regulated at 3 hpt, and all were maintained at low expression levels until 24 hpt, although three of which returned to their initial expression levels at 120 hpt. In addition, two *NnWRKYs* were initially significantly down-regulated at 6 hpt, but were later found to be significantly up-regulated at 120 hpt.

In summary, we found a total of 32, 34, and 28 *NnWRKYs* positively regulated by SA, JA, and submergence treatments, respectively, 14 of which were involved in responses to all the three treatments. In addition, a set of 6, 4, and 12 *NnWRKYs* were found to be negatively regulated by SA, JA, and submergence treatment, respectively (Table 2), while no *NnWRKY* was commonly involved in negative regulation of the above treatments.

### 2.7. NnWRKY40a and NnWRKY40b are Involved in Lotus Benzylisoquinoline Alkaloid Biosynthesis

We have identified previously two lotus WRKYs, NnWRKY40a and NnWRKY40b, potentially acting as regulators in lotus benzylisoquinoline alkaloid (BIA) biosynthesis [36]. Thus, we validate specially the transcriptional regulation abilities of these two proteins on lotus alkaloid biosynthesis.

**Figure 7:**
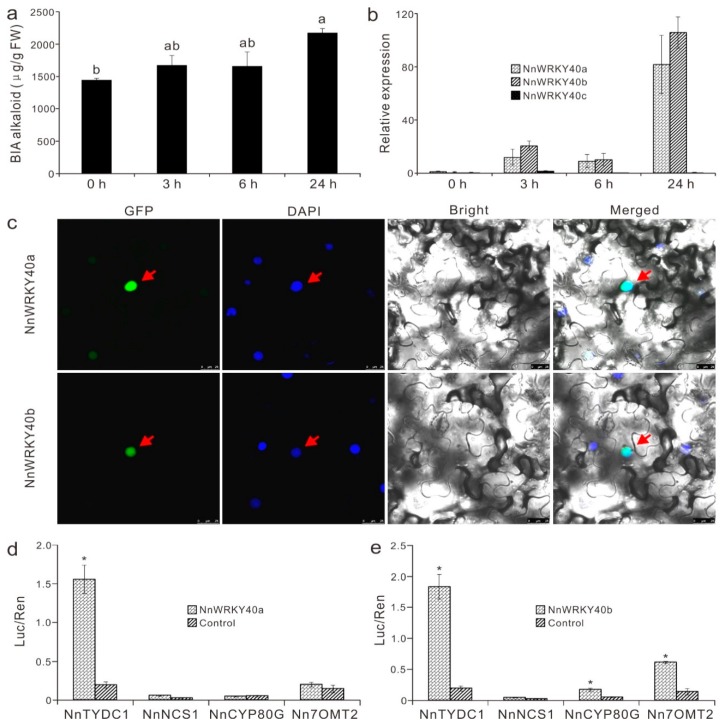
NnWRKY40a and NnWRKY40b are involved in lotus benzylisoquinoline alkaloid (BIA) biosynthesis. (**a**) BIA contents in lotus leaves treated by JA. Different lowercase letters indicate significant difference at *p* < 0.05. (**b**) Expression of *NnWRKY40a*, *NnWRKY40b* and *NnWRKY40c* in responses to JA treatment. (**c**) Subcellular localization of NnWRKY40a (upper-panel) and NnWRKY40b (lower-panel) in *Nicotiana benthamiana* leaves. Panels from left to right refer to GFP, DAPI, bright, and merged images respectively. Protein fusions co-localized with DAPI markers were marked with red arrows. (**d**,**e**) Estimation of the effect of NnWRKY40a and NnWRKY40b on activation of the promoter of BIA pathway genes using transient dual-luciferase assay. Error bars show SE of three biological replicates, and * represents significant difference at *p* < 0.05.

JA is well-known to induce the biosynthesis of various secondary metabolites including alkaloids, through activating a series of JA-responsive TFs [39]. Under the JA treatment, BIA content in lotus leaves was steadily increased, with a 15% increase at 3 and 6 hpt, and an approximately 50% increase at 24 hpt compared to the non-treated control (Figure 7a). *NnWRKY40a* and *NnWRKY40b* were significantly induced by the JA treatment, with their expressions at 3 hpt up-regulated to 11.85 and 30.56 times that in the leaf control, respectively. At 24 hpt, their expressions were dramatically up-regulated to 81.72 and 157.28 times that in the non-treated leaves, respectively (Figure 7b). *NnWRKY40c*, however, showed no response to JA treatment, and remained at low levels until 24 hpt. In addition, subcellular localization assay results showed that the 35S-drived fluorescent protein fusions of both NnWRKY40a:GFP and NnWRKY40b:GFP were located in the nucleus of the infiltrated *Nicotiana benthamiana* leaf cells (Figure 7c).

To verify whether NnWRKY40a and NnWRKY40b could bind to BIA biosynthetic gene promoters and drive their expressions, dual-luciferase assay was carried out, and promoters of four BIA biosynthetic genes, namely, *NnTYDC*, *NnNCS*, *NnCYP80G*, and *Nn7OMT*, were tested. Results showed that NnWRKY40a was able to activate the *NnTYDC* promoter (Figure 7d), and NnWRKY40b was well able to activate three BIA gene promoters, namely, *NnTYDC*, *NnCYP80G*, and *Nn7OMT* (Figure 7e). However, neither NnWRKY40a nor NnWRKY40b was able to activate the promoter of *NnNCS*. Taken together, both NnWRKY40a and NnWRKY40b can be induced by JA hormone treatment, and can activate one or more BIA biosynthetic genes to promote BIA biosynthesis.

## 3. Discussion

As one of the largest TF families in plants, the *WRKY* gene family has been identified in many terrestrial plant species, such as *Arabidopsis*, grape, rice, cucumber, tomato, pineapple, poplar, and tea plants [2,3,5,40,41,42,43,44], but is yet to be explored in the aquatic lotus plant. In this study, we report the genome-wide identification, classification, and expression analysis of WRKY family genes in lotus, and identified that NnWRKY40a and NnWRKY40b are involved in lotus BIA biosynthesis.

### 3.1. Characteristics of Lotus WRKY Gene Family

In total, we identified 65 *NnWRKY* genes in the 804 Mb lotus genome, which was relatively fewer than those reported in *Arabidopsis* (72), grape (59), tomato (81), rice (100), poplar (104), and soybean (176), considering their genome sizes and phylogenetic positions in the species tree [5,31,32,41,43,44]. NnWRKYs mostly contain the conserved WRKYGQK heptapeptide, while variants of WRKYGKK, WRKYSEK, WKKYGKK, and WKKYGQK were also observed. Similar cases have been reported in plant species such as *Arabidopsis*, grape, apple, and barley [45,46]. This variation in WRKY domain could alter their DNA binding affinity. Soybean GmWRKY6 and GmWRKY21 and tobacco NtWRKY12, for example, containing a WRKY domain of WRKYGKK, have been reported to bind the WK box (TTTTCCAC) rather than the W box [12,47]. The six NnWRKY proteins that harbor WRKYGQK variants thus might preferentially bind to non-W-box DNA *cis*-elements. Similar to their *Arabidopsis* homologs, NnWRKYs showed a consistent clustering pattern, forming seven groups designated I, IIa-IIe, and III. In addition, the group II NnWRKY members were distributed separately in several paraphyletic clusters, as has been observed in other species [5,6]. It should also be noted that, unlike *Arabidopsis* and grape, lotus does not have WRKY proteins clustered in the group IIIa, suggesting that members of this group in the eudicots might have evolved after the divergence of lotus from the core eudicots.

### 3.2. The Evolution Pattern of NnWRKY Genes

Both large- and small-scale gene duplications have been reported to play significant roles in the expansion of the *WRKY* gene family [43,48,49]. As has been previously proposed, two or more *WRKY* genes located within a 200-Kb chromosome region are deemed to be tandem duplicates [43]. Following this parameter, seven pairs of *NnWRKY* were found to be located in tandemly arrayed clusters: *NnWRKY6b* and a *NnWRKY9-*like gene (*NNU_24697*), *NnWRKY27a* and *NnWRKY43a* on Chr 1; *NnWRKY9* and *NnWRKY6a*, *NnWRKY55a* and *NnWRKY70a* on Chr 5; *NnWRKY55b* and *NnWRKY70b*, *NnWRKY53a* and *NnWRKY53b*, *NnWRKY40b* and *NnWRKY40c* on Chr 2, 3, and 8 respectively. In addition, seven weak tandem duplicate *WRKY* gene pairs within a 1000-Kb chromosomal area were observed in the lotus genome.

Synteny analysis of *NnWRKY* genes revealed that, of the 28 pairs of *NnWRKY*s, 26 pairs were in syntenic. These paired genes were highly similar not only in gene (or protein) sequences, but also in gene contents in the 2-Mbp chromosome regions around them. Another gene pair, *NnWRKY9* and *NNU_24697*, were also syntenic, and shared high NA (52.2%) and AA (57.7%) sequence similarity, although *NNU_24697* later lost its WRKY domain (Figure S1). These gene pairs were supposed to have evolved through a recent segmental duplication event. Meanwhile, syntenies between those non-paired *NnWRKY* genes, within or between phylogenetic groups, were quite frequently detected, suggesting that ancient segmental duplications contributed also to the expansion of lotus *WRKY* gene family.

In addition, *NnWRKY* evolution was also attributed to gene transpositions. *NnWRKY2a* and *NnWRKY2b*, for example, located in different chromosomes and with no syntenic relationship, are likely to be derived from small scale of duplications like transposition or retro-transposition. This was also the case for *NnWRKY21a* and *NnWRKY21b*. Moreover, syntenic chromosome regions that contain several tandemly arrayed syntenic *NnWRKY*s were also observed. For example, a fragment on Chr 3, containing four *NnWRKY* genes (*NnWRKY75b, NnWRKY72a, NnWRKY4a* and *NnWRKY57a*), corresponded well with a fragment on Chr 4 harboring the same set of genes. Eight more syntenic chromosome regions were also detected in this research, which included four pairs of chromosome blocks harboring 3 *WRKYs*, and four pairs of blocks harboring two *WRKYs* (Figure 3). These large chromosome duplication events observed in lotus are consistent with a recent study reported by Gui et al. [37], indicating a WGD event in lotus.

Lotus, as a basal eudicot, lacks the paleo-hexaploid (γ event) WGD, but has clearly experienced a very recent WGD approximately 18 Mya [50,51]. Following a WGD, most gene duplicates tend to quickly revert to their original singleton status due to extensive fractionations. However, those gene duplicates involved in gene transcription and signal transduction are preferentially retained [52,53,54]. Our analysis of *NnWRKY* gene duplications revealed that NnWRKY TFs involved in regulation of gene transcription were preferentially retained after the WGD, although domain gain and loss, for example in NNU_24697 and NnWRKY33a, and local tandem duplication in NnWRKY40b and NnWRKY40c exist and still occur. Thus, both large (WGD/segmental duplication) and small (tandem/gene translocation) scale gene duplication events seem to have shaped the current lotus *WRKY* gene family.

### 3.3. Roles of NnWRKYs on Responses to Biotic and Abiotic Stresses

SA and JA are major defense hormones modulating plant immune signaling networks [55,56]. SA is generally involved in local defense systems against biotrophic and hemi-biotrophic pathogens, as well as in systemic acquired resistance [57], whereas JA is required for the activation of plant defenses against nectrotrophic pathogens, and cooperates with ABA to induce plant defenses against herbivores [58]. Studies have shown that *WRKYs* are regulated by phytohormones like SA and JA, and play important roles in hormone-mediated signaling pathways [59]. In addition, lotus is an aquatic plant which suffers frequent submergence stress due to rapid increases in water levels. Thus, we subjected lotus leaves to SA and JA treatment, and plants to submergence stress. 

Overall, all *NnWRKYs* responded to at least one of the three treatments, indicating their extensive involvement in environmental adaptation. We found a set of 32, 34, and 28 *NnWRKYs* that were positively regulated, and a set of 6, 4, and 12 *NnWRKYs* that were negatively regulated by SA, JA, and submergence treatment, respectively. There were 14 *NnWRKY* genes that were commonly up-regulated by all the three treatments, although their response patterns varied. For example, *NnWRKY17a* was significantly up-regulated by SA treatment early at 3 hpt, but was not significantly up-regulated until 24 and 120 hpt after JA and submergence treatments, respectively. *NnWRKY* genes responded generally quickly to SA treatment, with their expressions mostly peaking at 3 hpt. In contrast, JA responsive *NnWRKYs* showed late peak expressions at 24 hpt, which is consistent with previous observations in *Arabidopsis* and pineapple [23,42]. In addition, the SA and JA-mediated signaling pathways were generally thought to act antagonistically [60,61], while possible synergistic interactions were also reported [62,63]. In this study, we identified a total of 26 *NnWRKY* genes that were up-regulated both by SA and JA treatments, indicating an intense synergistic interaction between SA and JA-mediated immune systems in lotus. 

### 3.4. Role of NnWRKYs in Regulation of Lotus Alkaloid Biosynthesis

JA and its derivatives can induce several types of TFs that participate in secondary metabolite biosynthesis, including AP2/ERF, bHLH, MYB, and WRKY [39]. It has been reported that *Catharanthus roseus* and *Coptis japonica* WRKYs, CrWRKY1 and CjWRKY1, both responded to JA signal, and regulated the biosynthesis of terpene indole alkaloid (TIA) and BIA through a JA-mediated signaling pathway [25,64]. A WRKY in *Papaver somniferum* could also be induced by wounding and exogenous MeJA, and regulate BIA synthesis [65]. We have previously isolated two BIA candidate regulators, NnWRKY40a and NnWRKY40b, through comparative RNA sequence analysis [36]. Both the expressions of *NnWRKY40a* and *NnWRKY40b*, and the BIA biosynthesis were dramatically induced by JA treatment. Dual-luciferase report gene assays proved that NnWRKY40a can truly activate *NnTYDC1* promoter, and NnWRKY40b can active three BIA biosynthetic gene promoters. Thus, JA-responsive lotus WRKYs, NnWRKY40a and NnWRKY40b, were both involved in regulation of lotus BIA biosynthesis. 

## 4. Materials and Methods

### 4.1. Plant Materials, Treatments, and Alkaloid Quantification

The lotus cultivar ‘BaiHuaJian’ (BJ) used in this study was grown in the lotus planting base at the Wuhan Botanical Garden of the Chinese Academy of Sciences (Wuhan, Hubei province, China). All tissue samples, including roots, rhizomes, leaves, petals, embryos, seedpods, petioles and stamens, were collected during the blossom season in the summer of 2018. Samples were immediately frozen in liquid nitrogen and stored in −80 °C until use. 

For the hormone assays, 5 mM SA or 0.1 mM methyl jasmonate (MeJA) solution was sprayed on lotus leaves at the 4th (S4) developmental stage [35]. Leaves sprayed with sterile water were used as a control. To prevent solution evaporation, treated leaves were wrapped with transparent plastic film. Leaf samples were taken at 0, 3, 6 and 24 h post treatment (hpt). For submergence treatment, half-year-old lotus plants were submerged in the water and leaves at the S4 stage were sampled at 3, 6, 24 and 120 hpt. As a control, leaves were taken from untreated pot plants.

Lotus alkaloids in MeJA treated leaves were extracted and quantified the same as in our previously reported protocol [35].

### 4.2. Mining of WRKY Genes

Nucleic and amino acid sequences of all AtWRKYs were downloaded from the *Arabidopsis* information resource (TAIR) database (https://www.arabidopsis.org/). To perform a genome-wide survey of all NnWRKYs, a text file that includes full-length protein sequences of all AtWRKYs was uploaded into the HMMER web server (https://www.ebi.ac.uk/Tools/hmmer/) as a query. The taxonomy was restricted on Nelumbo nucifera, and the cut-offs were set at an E-value of 0.01 and a hit of 0.03. Resulting sequences were subsequently examined for the presence of WRKY domains with the SMART online tool (http://smart.embl-heidelberg.de/). After removing redundant sequences and discarding those without WRKY domains, remaining proteins were deemed to be putative NnWRKYs. Finally, all NnWRKYs were taken as queries to BLAST against the genome database of ‘China Antique’ (ASM303368v1) [37], for identifying their full-length DNA sequences, coding DNA sequences (CDS), annotation IDs, and chromosome localizations.

### 4.3. Phylogenetic Analysis

A multiple sequence alignment was carried out with MUSCLE [66] based on the full-length protein sequences of lotus and *Arabidopsis* WRKYs. Neighbor-joining phylogenetic trees were constructed with MEGA7.0 software [67] with 1000 replicates. Phylogenetic trees were checked and modified with the software FigTree V1.4.2.

### 4.4. Analysis of WRKY Gene Structures and Conserved Domains

The exon–intron structures of *NnWRKY* genes were analyzed with the online program Gene Structure Display Server (GSDS, http://gsds.cbi.pku.edu.cn) [68], in which their coding sequences and full-length gene sequences were compared. To identify the conserved motifs in the NnWRKY proteins, the MEME online program (http://meme-suite.org/tools/meme) was used with the following parameters: minimum motif width = 6; maximum motif width = 50; maximum number of motif = 15; minimum sites per motif = 2; maximum sites per motif = 600 [69].

### 4.5. Synteny Analysis

Synteny analysis was conducted using the SynFind program in the CoGe platform [49]. Gene sequences of all *NnWRKYs* were each used as queries to search syntelogs in the lotus genome. Syntelogs are genomic regions that anchor to certain genes and contain similar gene content as that of the query gene. In this analysis, 1-Mbp genomic regions on either side of the query gene and target syntelogs were compared and the syntenic threshold was set at 4 per 100 genes.

### 4.6. RNA Extraction, Real-Time PCR and RNA Sequencing Analysis

Total RNA of lotus samples was extracted with a RNAprep Pure Plant Kit (Tiangen Biotec, Beijing, China), and reverse transcription was carried out using the cDNA Synthesis SuperMix (TransGen, Beijing, China) according to the manufacturer’s instructions. The quantitative real time PCR was performed on StepOnePlusTM Real Time PCR system (Applied Biosystems, Foster City, CA, USA) using SYBR^®^ Premix Ex Taq™ II (Takara, Dalian, China), with the lotus *ACTIN* as an internal control. The total reaction volume was set as 15 μL containing 3 μL of 10× diluted cDNA, 7.5 μL of 2× SYBR Green Master Mix and 0.2 μM of each primer (listed in Table S2). The relative gene expression was calculated with the ∆∆Ct method [70]. This experiment was carried out 2 times with 3 biological replicates. 

For SA-, JA- and submergence-treated plants, 3 leaf samples per time point were taken and submitted for RNA-seq library construction and sequencing by Biomarker Technologies Co, Ltd (Beijing, China). The digital expression data of all *NnWRKY* genes were then collected and analyzed with the online heat map program Morpheus (https://software.broadinstitute.org/morpheus).

### 4.7. Dual-Luciferase Report Gene Assay

Transient dual luciferase reporter assay was conducted in *Nicotiana benthamiana*, and the 4 firefly and *Renilla* luciferase reporter vectors carrying promoters of *NnTYDC1*, *NnNCS1*, *NnCYP80G*, and *Nn7OMT2*, respectively, were the same as have been used in our previous research [36]. Full length CDS of NnWRKY40a and NnWRKY40b were PCR amplified and inserted into pSAK277 vectors under the 35S promoter. *Agrobacterium* transformation, agro-infiltration, and luciferase activity assay were performed as previously described [36].

### 4.8. Subcellular Localization Assay

Subcellular localization assay of NnWRKY40a and NnWRKY40b was performed by transient expression in *Nicotiana benthamiana*. Full length CDS of *NnWRKY40a* and *NnWRKY40b* were cloned into pMDC83 vector individually to express the fluorescent protein fusions of NnWRKY40a:GFP and NnWRKY40b:GFP. For plant infiltration, we used 4- to 5-week-old *N. benthamiana* plants grown in the growth chamber with controlled environment conditions of day/night temperature of 24/22 °C and photoperiod of 16h light:8h dark. *Agrobacterium* strain GV3101 transformed with pMDC83 vectors was prepared the same as in the dual-luciferase report gene assay but was resuspended in the infiltration buffer to the final OD600 value as 0.4. Imaging was carried out 2 days after agroinfiltration, and the nucleus marker of DAPI solution (5μg/mL) was infiltrated to the same area 30 min before sampling. The images were acquired using a Zeiss fluorescence confocal microscope (LSM710 Meta, Carl Zeiss) with the following settings: DAPI was excited with a 358-nm (ultraviolet) laser and detected at 461 nm (blue); GFP was excited by a 488-nm laser and detected at 510 nm.

## 5. Conclusions

Lotus genome encodes a set of 65 *WRKY* genes, most of which fall functionally in pair. This attributes largely to the recently occurred whole genome duplication event in lotus. Lotus WRKYs involved intensively in SA- and JA-mediated responses to both biotic and abiotic stresses. Two lotus WRKYs, NnWRKY40a and NnWRKY40a, can activate one or more BIA biosynthetic gene expression and enhance the lotus BIA biosynthesis.

## Figures and Tables

**Table 1:** Nucleic acid (NA) and amino acid (AA) sequence similarities of functionally paired lotus WRKYs.

No	Lotus WRKY Pairs	NA Identity	AA Identity
1	NnWRKY2a and NnWRKY2b	54.8%	41.6%
2	NnWRKY4a and NnWRKY4b *	47.7%	44.8%
3	NnWRKY6a and NnWRKY6b *	73.0%	60.1%
4	NnWRKY7a and NnWRKY7b *	80.4%	79.5%
5	NnWRKY12a and NnWRKY12b *	81.6%	78.5%
6	NnWRKY13a and NnWRKY13b *	60.4%	53.9%
7	NnWRKY17a and NnWRKY17b *	56.9%	60.1%
8	NnWRKY20a and NnWRKY20b *	55.3%	50.9%
9	NnWRKY21a and NnWRKY21b	67.4%	57.5%
10	NnWRKY23a and NnWRKY23b *	78.7%	76.3%
11	NnWRKY27a and NnWRKY27b *	65.4%	62.4%
12	NnWRKY28a and NnWRKY28b *	80.2%	77.2%
13	NnWRKY31a and NnWRKY31b *	76.2%	76.6%
14	NnWRKY32a and NnWRKY32b *	73.9%	70.0%
15	NnWRKY33a and NnWRKY33b *	69.8%	64.3%
16	NnWRKY43a and NnWRKY43b *	78.6%	75.2%
17	NnWRKY44a and NnWRKY44b *	82.0%	75.6%
18	NnWRKY49a and NnWRKY49b *	80.0%	74.8%
19	NnWRKY50a and NnWRKY50b *	63.6%	64.2%
20	NnWRKY51a and NnWRKY51b *	70.5%	72.5%
21	NnWRKY53a and NnWRKY53b	90.4%	86.0%
22	NnWRKY55a and NnWRKY55b *	54.4%	43.2%
23	NnWRKY57a and NnWRKY57b *	79.4%	78.0%
24	NnWRKY65a and NnWRKY65b *	78.8%	72.1%
25	NnWRKY69a and NnWRKY69b *	77.6%	66.9%
26	NnWRKY70a and NnWRKY70b *	70.8%	63.7%
27	NnWRKY72a and NnWRKY72b *	77.3%	73.0%
28	NnWRKY75a and NnWRKY75b *	81.9%	75.4%
29	NnWRKY40a and NnWRKY40b/c *	75.6%/45.0%	73.8%/42.0%
30	NnWRKY9 and NNU_24697 *	52.2%	57.7%

Note: Functionally paired genes marked with “*” are syntenic. NnWRKY53a and NnWRKY53b are tandemly arrayed on Chr3. NnWRKY40a, NnWRKY40b, and NnWRKY40c form functionally an NnWRKY triad, and ‘NNU_24697′ is a paralogue of NnWRKY9 that lacks a WRKY domain.

**Table 2:** *NnWRKY*s that are positively or negatively regulated by SA, JA, and submergence (SM) treatment.

Treatments	Positively Regulated *WRKYs*	Negatively Regulated *WRKYs*
SA	32 *NnWRKY*s: *NnWRKY4aNnWRKY7a NnWRKY7b NnWRKY17a NnWRKY23a NnWRKY27aNnWRKY28aNnWRKY28bNnWRKY31a NnWRKY31b WRKY33a NnWRKY33b NnWRKY40a NnWRKY40b NnWRKY41 NnWRKY43b NnWRKY47 NnWRKY49a NnWRKY49b NnWRKY50a NnWRKY50b NnWRKY51a NnWRKY51b NnWRKY55b NnWRKY65a NnWRKY70a NnWRKY70b NnWRKY72a NnWRKY72b NnWRKY75a NnWRKY75b Nnu_23618*	6 *NnWRKY*s: *NnWRKY6a NnWRKY12b NnWRKY23b NnWRKY44a NnWRKY55a NnWRKY69a*
JA	34 *NnWRKYs*: *NnWRKY2b NnWRKY4a NnWRKY6a NnWRKY7a NnWRKY7b NnWRKY17a NnWRKY23a NnWRKY23b NnWRKY27a NnWRKY27b NnWRKY28aNnWRKY28bNnWRKY31a NnWRKY31b NnWRKY33a NnWRKY33b NnWRKY40a NnWRKY40b NnWRKY41 NnWRKY47 NnWRKY50a NnWRKY50b NnWRKY51a NnWRKY51b NnWRKY53a NnWRKY53b NnWRKY57a NnWRKY57b NnWRKY65a NnWRKY70a NnWRKY70b NnWRKY72aNnWRKY75a NnWRKY75b*	4 *NnWRKY*s: *NnWRKY12a NnWRKY40c NnWRKY49b NnWRKY69a*
SM	28 *NnWRKYs*: *NnWRKY4a NnWRKY4b NnWRKY6a NnWRKY7a NnWRKY12a NnWRKY17a NnWRKY21a NnWRKY23b NnWRKY27a NnWRKY27b NnWRKY28aNnWRKY28bNnWRKY31aNnWRKY33a NnWRKY43b NnWRKY49a NnWRKY49b NnWRKY50a NnWRKY50b NnWRKY55a NnWRKY55b NnWRKY57b NnWRKY65a NnWRKY69b NnWRKY72a NnWRKY72b NnWRKY75a NnWRKY75b *	12 *NnWRKY*s: *NnWRKY9 NnWRKY12b NnWRKY23a NnWRKY40a NnWRKY40b NnWRKY41 NnWRKY44b NnWRKY51a NnWRKY51b NnWRKY53a NnWRKY53b Nnu_23618*

**Note:***NnWRKYs* marked with red were commonly involved in the responses to all three treatments.

## Supplementary Materials

Supplementary materials can be found at https://www.mdpi.com/1422-0067/20/20/5006/s1.
