# Supplementary material for: Comprehensive Analysis and Functional Studies of WRKY Transcription Factors in Nelumbo nucifera
Source: Int J Mol Sci. 2019 Oct 10;20(20):5006. doi: 10.3390/ijms20205006 (PMC6829473; doi:10.3390/ijms20205006)
Supplement: Supplementary file 1 [file ijms-20-05006-s001.pdf]

Table S1 List of WRKY family members identified genome-wide in *Nelumbo nucifera*.

Gene Name	Annotation/Genbank ID	Chrom-osome	Location	ORF Length (Nts)	Group	WRKY Domain	Zinc-finger Type	Note
NnWRKY2a	NNU_20595/XM_010254575	1	60331779-60325443	2232	I	WRKYGQK/WRKYGQK	C2H2	
NnWRKY2b	NNU_17962/XR_736581	1	6381110-6371515	2358	I	WRKYGQK/WRKYGQK	C2H2	
NnWRKY4a	NNU_07349/XM_010260422	3	93380654-93385304	1617	I	WRKYGQK/WRKYGQK	C2H2	
NnWRKY4b	NNU_04581/XM_010259913	4	73164730-73182056	2694	I	WRKYGQK/WRKYGQK	C2H2	
NnWRKY6a	NNU_16107/XM_019196196	5	46697578-46699727	1512	IIb	WRKYGQK	C2H2	
NnWRKY6b	NNU_00165/XM_010248058	1	30869961-30867814	1512	IIb	WRKYGQK	C2H2	
NnWRKY7a	NNU_13622/XM_010272500	5	9965414-9966974	1038	IId	WRKYGQK	C2H2	
NnWRKY7b	NNU_17024/XM_010265142	1	117568068-117566457	1077	IId	WRKYGQK	C2H2	
NnWRKY9	NNU_16105/XM_010246787	5	46661891-46663927	1581	IIb	WRKYGQK	C2H2	
NnWRKY12a	NNU_14361/XM_010260434	6	35217906-35210883	651	IIc	WRKYGQK	C2H2	
NnWRKY12b	NNU_16551/XM_010247799	1	200462041-200470342	669	IIc	WRKYGQK	C2H2	
NnWRKY13a	NNU_20076/XM_010262725	5	32536749-32539936	672	IIc	WRKYGQK	C2H2	
NnWRKY13b	NNU_06423/XM_010270288	1	91952526-91946637	477	IIc	WRKYGQK	C2H2	
NnWRKY14	NNU_08414/XM_010249539	7	18757624-18759965	1305	IIe	WRKYGQK	C2H2	
NnWRKY17a	NNU_14734/XM_010267113	8	32431912-32433307	972	IId	WRKYGQK	C2H2	
NnWRKY17b	newGene_16312/XM_010258956	1	158699740-158700138	312	IId	WRKYGQK	/	No Zinc-finger structure
NnWRKY20a	NNU_16003/XM_010271865	1	148596912-148607860	1482	I	WRKYGQK/WRKYGQK	C2H2	No Zinc-finger structure in the C-terminal WRKY domain
NnWRKY20b	NNU_20475/XM_010254390	1	64157925-64156753	972	I	WRKYGQK/WRKYGQK	C2H2	No Zinc-finger structure in the C-terminal WRKY domain
NnWRKY21a	NNU_19902/XM_010279826	1	162954901-162952926	1083	IId	WRKYGQK	C2H2	
NnWRKY21b	NNU_14264/XM_010279826	3	60701675-60700616	915	IId	WRKYAQK	/	No Zinc-finger structure
NnWRKY23a	NNU_05834/XM_010269637	4	21149116-21147855	933	IIc	WRKYGQK	C2H2	
NnWRKY23b	NNU_13849/XM_010260939	3	3896431-3897619	900	IIc	WRKYGQK	C2H2	

NnWRKY27a	NNU_01367/XM_010263082	1	180154804-180153481	1098	IIe	WRKYGQK	C2H2	
NnWRKY27b	NNU_04335/XM_010280754	6	17631405-17630060	867	IIe	WRKYGQK	C2H2	
NnWRKY28a	NNU_06594/XM_010277346	4	4102930-4101049	966	IIc	WRKYGQK	C2H2	
NnWRKY28b	NNU_19178/XM_010245403	3	22658175-22659903	984	IIc	WRKYGQK	C2H2	
NnWRKY31a	NNU_20314/XM_010254164	1	68179403-68181856	1842	IIb	WRKYGQK	C2H2	
NnWRKY31b	NNU_24624/XM_010246172	1	144761011-144747256	2058	IIb	WRKYGQK	C2H2	
NnWRKY32a	NNU_12096/XM_010267283	2	78178981-78166858	1698	I	WRKYGQK/WRKYGQK	C2H2	
NnWRKY32b	NNU_19344/XM_010277517	8	21729170-21740338	1827	I	WRKYGQK/WRKYGQK	C2H2	
NnWRKY33a	NNU_18828/XM_010258703	2	98850785-98853547	1767	I	WRKYGQK	C2H2	Only one WRKY domain available
NnWRKY33b	NNU_13013/XM_010266210	2	63037802-63040904	1977	I	WRKYGQK/WRKYGQK	C2H2	
NnWRKY40a	NNU_12117/XM_010267315	2	78900468-78899025	1050	IIa	WRKYGQK	C2H2	
NnWRKY40b	NNU_19326/XM_010277476	8	21258631-21260028	942	IIa	WRKYGQK	C2H2	
NnWRKY40c	newGene_3441/XM_019199981	8	21285537-21286840	714	IIa	WRKYGQK	C2H2	
NnWRKY41	NNU_01465/XM_010263210	1	177536134-177537992	1089	IIIb	WRKYGQK	C2HC	
NnWRKY43a	NNU_01372/XM_010263088	1	180041789-180035669	597	IIc	WRKYGQK	C2H2	
NnWRKY43b	newGene_98/XM_010248991	6	21869050-21857863	615	IIc	WRKYGQK	C2H2	
NnWRKY44a	NNU_12426/XM_010255152	2	105416670-105419110	1386	I	WRKYGQK/WRKYGQK	C2H2	
NnWRKY44b	NNU_15232/XM_010261834	2	68034522-68036671	1422	I	WRKYGQK/WRKYGQK	C2H2	
NnWRKY47	NNU_08253/XM_010281369	4	30139432-30133355	1494	IIb	WRKYGQK	C2H2	
NnWRKY49a	NNU_18755/XM_010258584	2	96951968-96956010	912	IIc	WRKYGQK	C2H2	
NnWRKY49b	NNU_03597/XM_010264244	2	61257443-61259593	909	IIc	WRKYGQK	C2H2	
NnWRKY50a	NNU_11881/XM_024052442	8	3882291-3886495	318	IIc	WKKYGKK	C2H2	
NnWRKY50b	NNU_05136/XM_030592689	6	63880471-63877082	615	IIc	WKKYGKK	C2H2	
NnWRKY51a	NNU_20102/XM_010262768	5	31909752-31907769	387	IIc	WRKYGKK	C2H2	
NnWRKY51b	NNU_09891/XM_010245010	1	109917294-109916003	321	IIc	WRKYGKK	C2H2	
NnWRKY53a	NNU_02487/XM_010268162	3	68565759-68563758	1092	IIIb	WRKYGQK	C2HC	

NnWRKY53b	NNU_02488/XM_010268164	3	68581263-68579174	1089	IIIb	WRKYGQK	C2HC
NnWRKY55a	NNU_24386/XM_010254110	5	78045097-78049321	1068	IIIb	WRKYGQK	C2HC
NnWRKY55b	newGene_5274/XM_020834403	2	109823115-109826669	1083	IIIb	WRKYGQK	C2HC
NnWRKY57a	NNU_07383/XM_010260920	3	93002824-92999921	936	IIc	WRKYGQK	C2H2
NnWRKY57b	NNU_04527/XM_010259818	4	72347210-72344413	927	IIc	WRKYGQK	C2H2
NnWRKY65a	NNU_25670/XM_010250060	6	47741891-47740244	912	IIe	WRKYGQK	C2H2
NnWRKY65b	NNU_16122/XM_010243807	3	16520969-16522637	891	IIe	WRKYGQK	C2H2
NnWRKY69a	NNU_13879/XM_010260989	3	4539392-4537925	849	IIe	WRKYGQK	C2H2
NnWRKY69b	NNU_05863/XM_010269687	4	20498200-20499825	86	IIe	WRKYGQK	C2H2
NnWRKY70a	NNU_24385/XM_010253742	5	78069123-78067671	981	IIIb	WRKYGQK	C2HC
NnWRKY70b	NNU_12194/XM_010254742	2	109846840-109845383	990	IIIb	WRKYGQK	C2HC
NnWRKY72a	NNU_07209/XM_010258267	3	96330092-96327476	1731	IIb	WRKYGQK	C2H2
NnWRKY72b	NNU_07819/XM_010244080	4	76498712-76495223	1869	IIb	WRKYGQK	C2H2
NnWRKY75a	NNU_22208/XM_010250769	4	87706954-87704421	555	IIc	WRKYGQK	C2H2
NnWRKY75b	NNU_02028/XM_010252352	3	106232273-106229307	552	IIc	WRKYGQK	C2H2
/	NNU_04803/XM_010257999	5	49193574-49201749	2061	/	WRKYSEK	C2HC
/	NNU_23618/XM_010278098	2	1018601-1025115	381	/	WKKYGQK	C2H2

Note: The 6 WRKY domains marked with red captital letters are those different to the conserved “WRKYGQK” domain.

Table S2 Primers used for in the research.

Gene name	Forward primer (5'-3')	Reverse primer (5'-3')	Application
NnWRKY2a	CTGAAAGAAGTAAGGATGGCACT	CTTCCCTGATTGAACTGAGACC	qRT-PCR
NnWRKY2b	TCAAATGGCTTCTATTGCTCAG	TGTATCAGTTTCCTTGGGTGG	qRT-PCR
NnWRKY4a	GTTTACGATTCCGCCTGGTTT	GAGGTGCTGGCACTGAAGATG	qRT-PCR
NnWRKY4b	CCCTTTAGTATCAATGGACCTTC	ACTCACTTCCTTTCACCTGCTT	qRT-PCR
NnWRKY6a	AATTCCGCCTTCCTAGCTCTT	TCTTCAACCTTCCAGTCCCTC	qRT-PCR
NnWRKY6b	TTGACCTGACCAACAACCCAC	ACTGCTTCTCATCCATGCCTC	qRT-PCR
NnWRKY7a	ACATCCAACACCTCCCTCCTC	GCTGCTTGCTATCAGTATCACCC	qRT-PCR
NnWRKY7b	AGAGGCAACAGCAGCAACACC	GGGAGGAGAACTACCCAAAGGAC	qRT-PCR
NnWRKY9	GTTCTTCACATTCACAGCCACA	CTTCTTCCGTCTTCCAGTTGC	qRT-PCR
NnWRKY12a	CCTCTTTGGAATGACACCCAG	CTAGCACATCCACATCGCTTC	qRT-PCR
NnWRKY12b	TGGGCTTAGTGGCGACGATGT	TTCCTCCTCACCTTTACCTTG	qRT-PCR
NnWRKY13a	TCTTCGGTGCAAAGATCGGCTAA	ACCTGGGCTCCCTCACCTTCCT	qRT-PCR
NnWRKY13b	GGGTACAAGACAGTGAGCGTTTG	ACCATCGTCCAAGACATCCAC	qRT-PCR
NnWRKY14	TTTAGCAGACATAGTGCGTGGTG	GGTAGCATAGGATCTCGCGTAG	qRT-PCR
NnWRKY17a	CTCATCAGAGCCCTCAACACCA	GGAACCGAGAAGGACGTAGAAGC	qRT-PCR
NnWRKY17b	TTAGCCAGAGTCTCGCACGGAT	ACTGACTTGCCAACAGCATCCA	qRT-PCR
NnWRKY20a	TAAAGGCTTCCACAGGTTTGA	GGGTCCGGTGCAGTTACAGAT	qRT-PCR
NnWRKY20b	CTCACTCATTTGCAGCCTTAT	AAATACCTGGAACCCACTATCA	qRT-PCR
NnWRKY21a	CCCAGTTCAGGAGATGGTTAC	GGTGTTGGAGGAATTGATAGTGT	qRT-PCR
NnWRKY21b	AGGAGGTTGAGGAAACTAACAG	TTAAGACCCAATCCAATACCA	qRT-PCR
NnWRKY23a	CGGAGTCGTCTGAGGTGTTGA	CTCTGCCGTTTCTGGTTCTTTT	qRT-PCR
NnWRKY23b	GCAACCTTCGATGCCATCACC	GTCTTCTCCTGCTCCTCCTCTT	qRT-PCR
NnWRKY27a	GCCACTACTACCACTGCACATTCTCA	GCGAGCCCTTGATTGGTTTCT	qRT-PCR
NnWRKY27b	AGAGGGTTGTTTGCCAGGTGC	TACGGCTGCGTTCGACTTGTT	qRT-PCR
NnWRKY28a	ACTACTGACATTCCGGCGACAC	TTTGGGCTTGCTCACTTTCTTATT	qRT-PCR
NnWRKY28b	CGGGTCATGGGCTAGGAGACT	CGGCATCATCCACAGGGTAAA	qRT-PCR
NnWRKY31a	ACCTCAGCGGCAGCTTGTATG	CCAATGTGACAGTCGGAAACG	qRT-PCR
NnWRKY31b	GACGACGGGATTTCACCTAATG	TTTCAGCCTTGCGGTTCTGTT	qRT-PCR
NnWRKY32a	TATGGTTACGAAGACACCCACT	ATTTCAGTCACAAAGCCAGTATG	qRT-PCR
NnWRKY32b	AAGGATCAAGGATAGCGATACAG	AGGATTTCCCTTCACCATTTT	qRT-PCR
NnWRKY33a	ATCACCACTTACGAGGGCAAAC	TGGAGCTGTAGGGACTAAAGGTAT	qRT-PCR
NnWRKY33b	GGATGGACAGATAACCGAGATAGT	TGCGAATGATTGGTCAGGGAT	qRT-PCR
NnWRKY40a	GGGGAGGAATCACTCGGTAAA	TGAAGCCCGTTTCGTTGTTCT	qRT-PCR
NnWRKY40b	AAGATGCTAGTGTACTGGTGGAGA	CAGGGAAGTTCGTTTCGTTATT	qRT-PCR
NnWRKY40c	ACTGAGGCAGATACAAGAAAGGGG	TGCAAAAGGATTAACTGTGGGAGA	qRT-PCR
NnWRKY41	AGTTCCAGCCTCGGCATCTTC	CATTGTCCAGGTCCTCGGTTT	qRT-PCR
NnWRKY43a	GGAAATAAAGCCGAAGATGAAA	GCGATAATAACTCCTGGGAAAG	qRT-PCR
NnWRKY43b	ATTCCCAAATCATACCCGACAT	GCTCGCTCTTGCCTTATCTTTAC	qRT-PCR
NnWRKY44a	TCCCAGTTCATCTTACTTGCC	TTCGCTTACATCTTAGTTCATTGTC	qRT-PCR
NnWRKY44b	GCCAGAAGGTTGTGAAGGGAA	GCAATGGGCATCTCGTGGTTA	qRT-PCR
NnWRKY47	ATGGTCGAGACCGTAACTGCG	TTGTGGTGATTCAGGCGATGC	qRT-PCR

NnWRKY49a	TACACCACCAAGCCCATTTAG	CAAGAAAGGGTAGAAGAGGAAGA	qRT-PCR
NnWRKY49b	CCATCTTACCAGGCTTACCAA	TCGGCTCACATCTCCTTCTAC	qRT-PCR
NnWRKY50a	GATATTATGGACGACGGCTTCA	GTTGTGGGTGCCTTCGTATGT	qRT-PCR
NnWRKY50b	CCCATCATCTCCTAAACTTCG	TTCAGCTTGACTGCCATTCTT	qRT-PCR
NnWRKY51a	GGACCGAGAAGATTCGAGCTA	CAAGAGGAAGATGAATTCGAAGC	qRT-PCR
NnWRKY51b	AGACCGTGAGGACTCGAGGTA	GATGATGAGTGTGAATGTGAAGC	qRT-PCR
NnWRKY53a	TGGCTTAGACTCTGGGGAGATGATG	GTAGTTGGATTCGGATGTTGCA	qRT-PCR
NnWRKY53b	CGATTTAGACTCTAGGGAGATGATA	GCAGTTGGATTCGGATGTTGCT	qRT-PCR
NnWRKY55a	ATGTATAGGGACTTTGACATGGGTAG	TTCTCGTCGATGGAAGGGAAG	qRT-PCR
NnWRKY55b	GACAGAAGGAAATTCTTGGGTCA	ACCTGATGATGGTGCTGTGGA	qRT-PCR
NnWRKY57a	CCCCACAGTTGTATTACCCAC	CAGGCACAATATCACCAAGCAGA	qRT-PCR
NnWRKY57b	TCACAACATACGAAGGCCAGCAT	GACATCAATCCCACCTCCCAGA	qRT-PCR
NnWRKY65a	CCACCACTTTCGCCAATTCCA	TCGCCCAGATCACCGAACTTATT	qRT-PCR
NnWRKY65b	CCGCTTCTCATTCTAACACCACC	TCTCCCAGATCACCGAACTTACTATC	qRT-PCR
NnWRKY69a	TAGGTGCAGCACATCTAAGGG	TTGTAGTATGGTGGCAGGGAG	qRT-PCR
NnWRKY69b	TGGAGGGCAGAAGAATGAAGG	GGTTGTGGGTGGAGGTGTAGG	qRT-PCR
NnWRKY70a	CAGGAATGCAAAGAGGTGGAGA	ACCTGAGTACACGCCCGAGAT	qRT-PCR
NnWRKY70b	GCCAGGAAGAAATCCCAAACC	ACGAGTAGACGCCCGAAATCA	qRT-PCR
NnWRKY72a	CAATCACATCAATCGTAGGTGGCG	AGCTCGATGCACATCCGTTCC	qRT-PCR
NnWRKY72b	CGACGACCCAGGGAAATCAAG	ATTCGACGAGGCCGATCTGTT	qRT-PCR
NnWRKY75a	GGGGAGAAGAAGATTAGAAAGC	TTGGATAGCCGTTGAACTTGC	qRT-PCR
NnWRKY75b	GGCAGCAGTAGCTGTTCCATT	TGATACCGAGCTTCGTTTCAC	qRT-PCR
NNU_04803	GTTTCCGTCGTCTTGTTGTCT	ACCTTCTCCTCGTAACCTTGA	qRT-PCR
NNU_23618	AGCTGAGGAAGGGTTCAGAGAA	GTTGAGATGTGGAGGAGGGATG	qRT-PCR
NnACTIN	CTCCGTGTTGCCCCTGAAG	CCAGCAAGGTCCAACCGAAG	qRT-PCR
NnWRKY44a	CGGAATTCATGCCGGCCATCGATCAATTT	GCTCTAGATCACCATTTTTGCACTAGTGAATT	Dual-luciferase assay
NnWRKY44b	CGGAATTCATGGAGTCGACTTGGTTGGAT	GCTCTAGATCACCATTTCTGCACTGTTGAATG	
NnWRKY44a	CCTTAATTAAATGCCGGCCATCGATCAATTT	TTGGCGCGCCACCATTTTTGCACTAGTGAATT	Subcellular localization
NnWRKY44b	CCTTAATTAAATGGAGTCGACTTGGTTGGAT	TTGGCGCGCCACCATTTCTGCACTGTTGAATG	

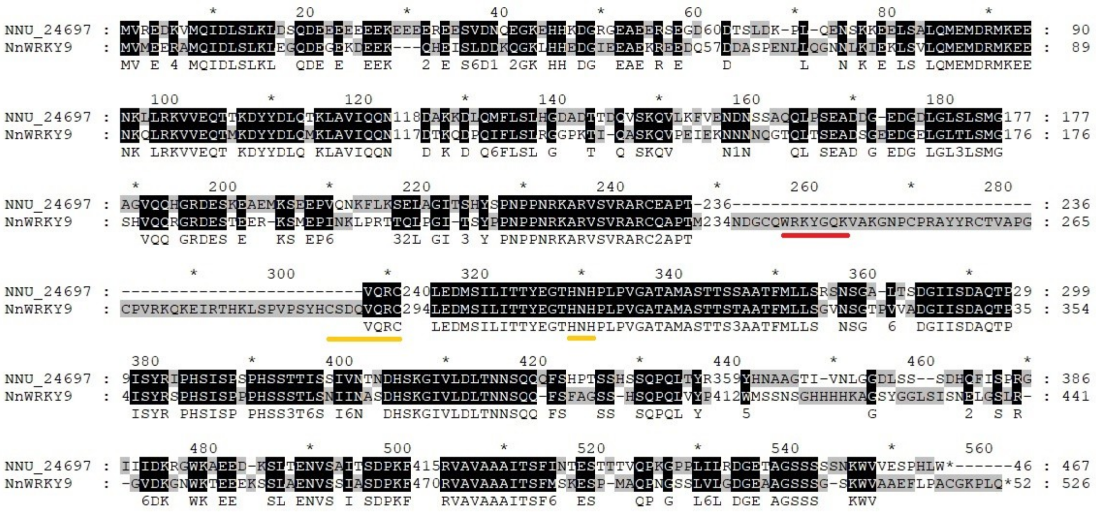

Fig. S1 Amino acid sequence alignment of NNU_24697 and NnWRKY9. Conserved WRKY domains were marked with red lines, and conserved zinc finger domains were marked with yellow lines.

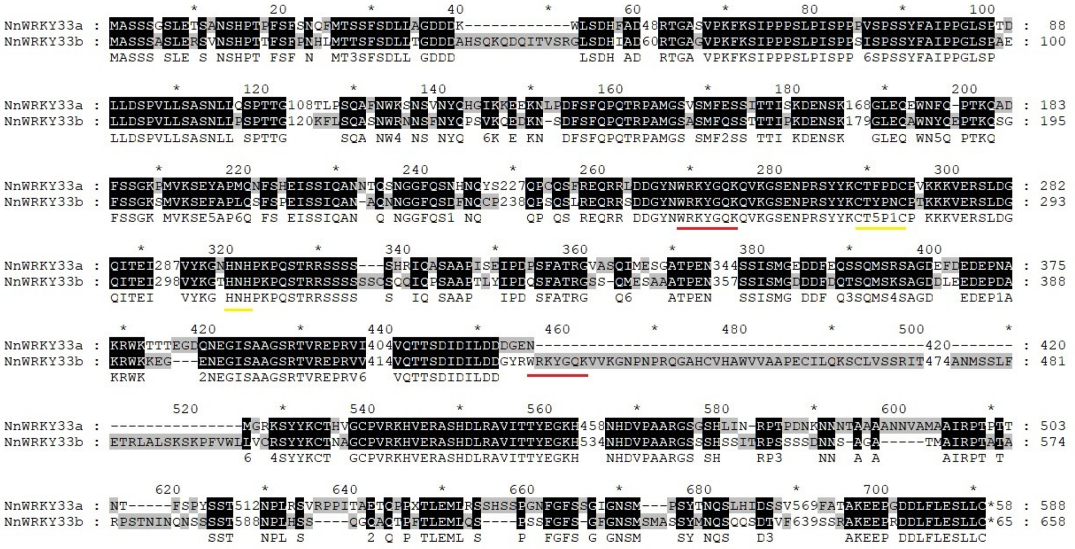

Fig. S2 Amino acid sequence alignment of NnWRKY33a and NnWRKY33b. Conserved WRKY domains were marked with red lines, and conserved zinc finger domains were marked with yellow lines.

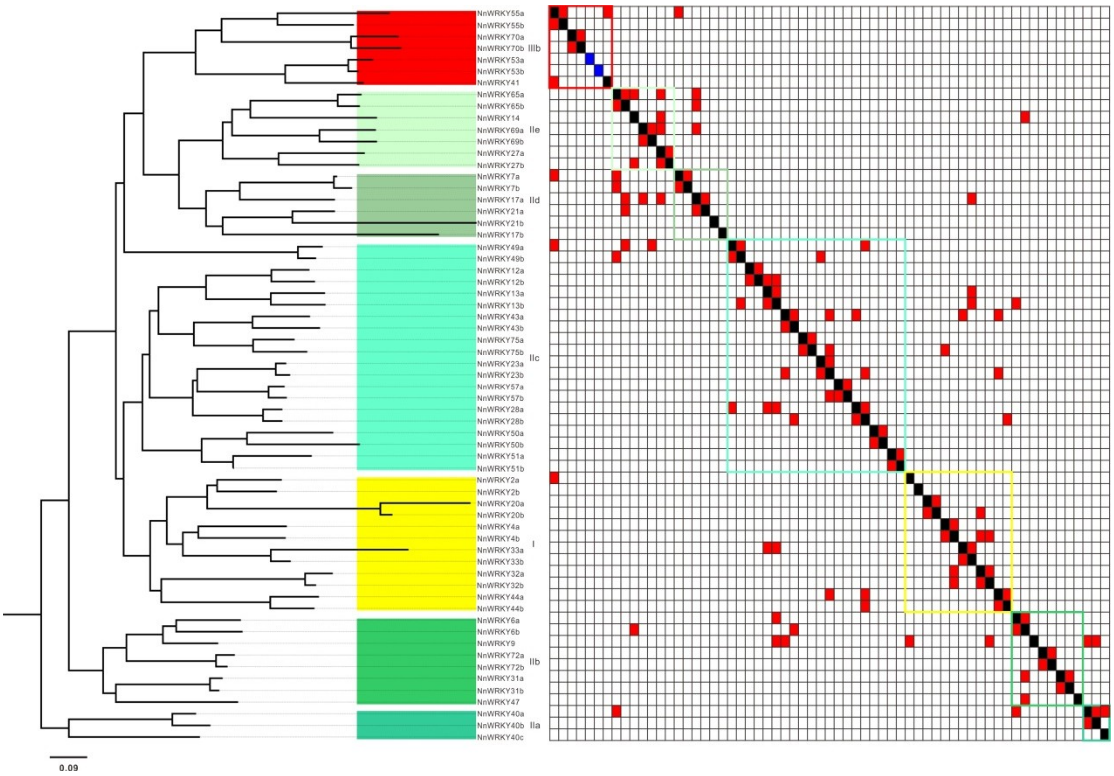

Fig. S3 Synteny analysis for lotus (*Nelumbo nucifera*) *WRKY* genes. Phylogenetic tree on the left panel was based on the full length protein sequences of 63 lotus WRKYs, and was made with neighbor-joining method in MEGA7 with 1000 bootstrap replications. Clusters of different phylogenetic groups were marked with different colors. Genes marked with black boxes were queries used for searching lotus *WRKY* syntelogs (syntenic homologs, marked with red boxes). Genes of a phylogenetic group were framed in the same color as its cluster.

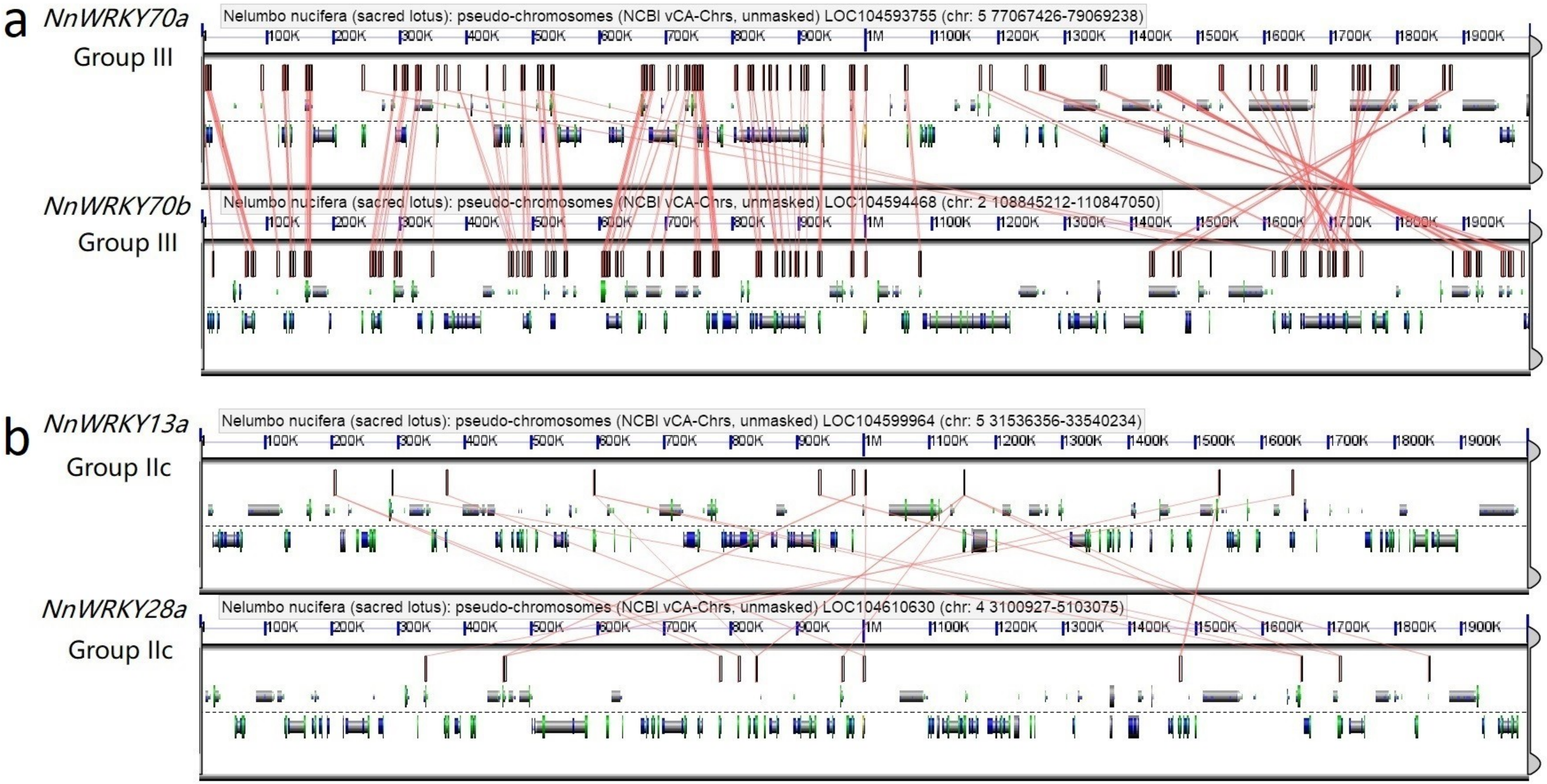
a
NnWRKY70a
Group III
Nelumbo nucifera (sacred lotus): pseudo-chromosomes (NCBI vCA-Chrs, unmasked) LOC104593755 (chr: 5 77067426-79069238)
NnWRKY70b
Group III
Nelumbo nucifera (sacred lotus): pseudo-chromosomes (NCBI vCA-Chrs, unmasked) LOC104594468 (chr: 2 108845212-110847050)
b
NnWRKY13a
Group IIc
Nelumbo nucifera (sacred lotus): pseudo-chromosomes (NCBI vCA-Chrs, unmasked) LOC104599964 (chr: 5 31536356-33540234)
NnWRKY28a
Group IIc
Nelumbo nucifera (sacred lotus): pseudo-chromosomes (NCBI vCA-Chrs, unmasked) LOC104610630 (chr: 4 3100927-5103075)
100K
200K
300K
400K
500K
600K
700K
800K
900K
1M
1100K
1200K
1300K
1400K
1500K
1600K
1700K
1800K
1900K

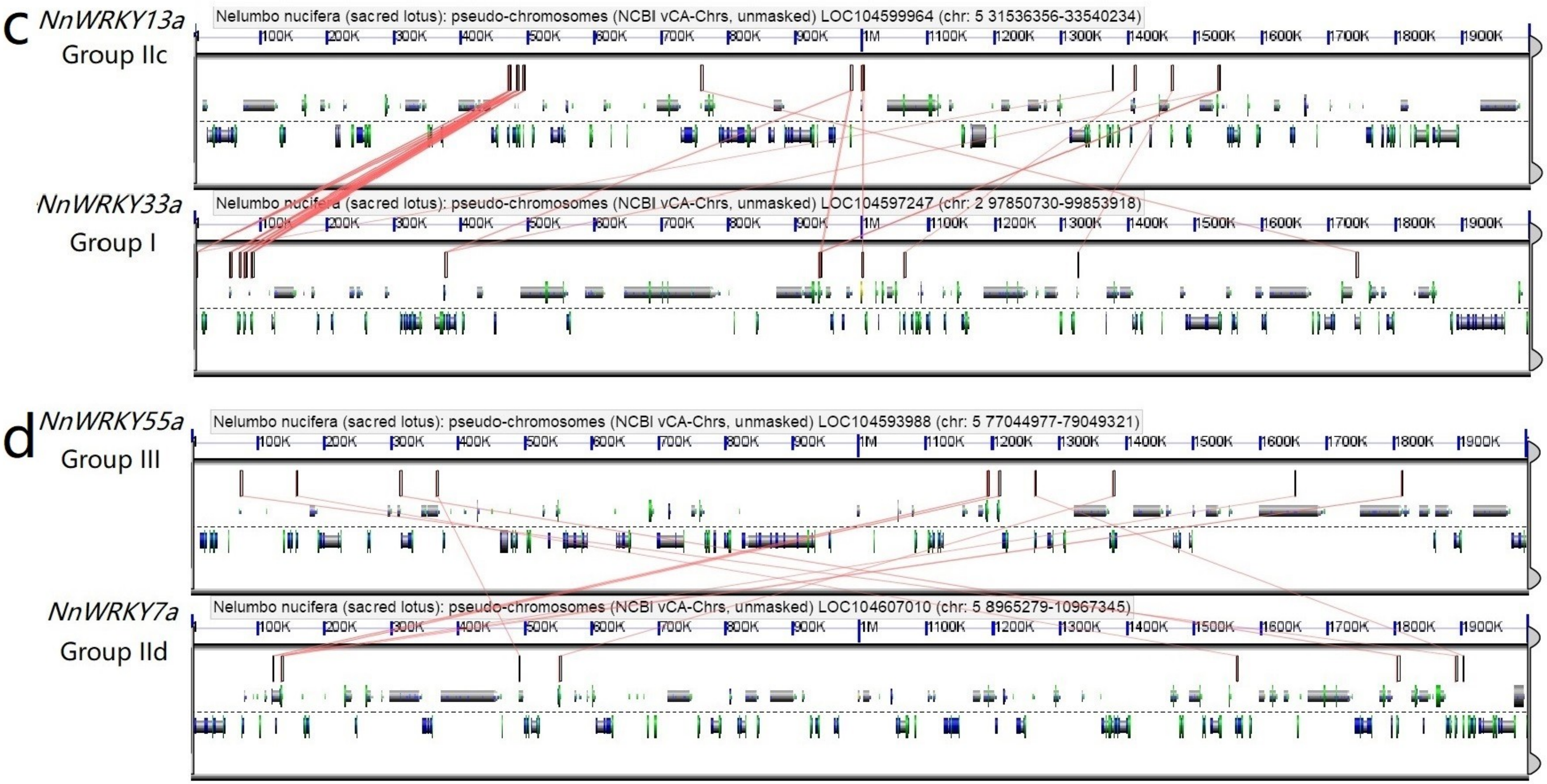
c
NnWRKY13a
Group IIc
Nelumbo nucifera (sacred lotus): pseudo-chromosomes (NCBI vCA-Chrs, unmasked) LOC104599964 (chr: 5 31536356-33540234)
NnWRKY33a
Group I
Nelumbo nucifera (sacred lotus): pseudo-chromosomes (NCBI vCA-Chrs, unmasked) LOC104597247 (chr: 2 97850730-99853918)
d
NnWRKY55a
Group III
Nelumbo nucifera (sacred lotus): pseudo-chromosomes (NCBI vCA-Chrs, unmasked) LOC104593988 (chr: 5 77044977-79049321)
NnWRKY7a
Group IId
Nelumbo nucifera (sacred lotus): pseudo-chromosomes (NCBI vCA-Chrs, unmasked) LOC104607010 (chr: 5 8965279-10967345)
100K
200K
300K
400K
500K
600K
700K
800K
900K
1M
1100K
1200K
1300K
1400K
1500K
1600K
1700K
1800K
1900K

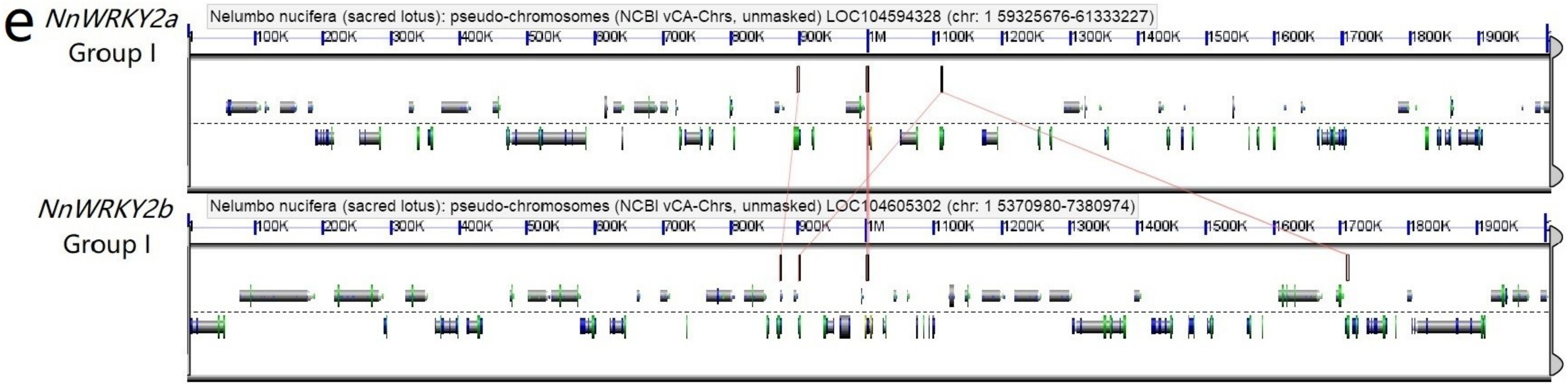

Fig. S4. Microsynteny analysis for representative lotus NnWRKY genes. (A) functionally paired genes with syntenic relationship; (B) syntenic genes within group IIc; (C) syntenic genes from group IIc and group I respectively; (D) syntenic genes from group III and group IId respectively; and (E), functionally paired genes with no syntenic relationship.
